# Editorial: The role of dietary interventions in the regulation of host-microbe interactions, volume II

**DOI:** 10.3389/fcimb.2025.1549914

**Published:** 2025-02-03

**Authors:** Xinyi Zhang, Yujie Cai, Xia He, Tingtao Chen

**Affiliations:** ^1^ School of Pharmacy, Jiangxi Medical College, Nanchang University, Nanchang, China; ^2^ Department of Obstetrics and Gynecology, The Ninth Hospital in Nanchang, Nanchang, China; ^3^ Queen Mary School, Jiangxi Medical College, Nanchang University, Nanchang, China

**Keywords:** dietary interventions, intestinal microbiota homeostasis, dysbiosis, probiotics/prebiotics/synbiotics, diabetes, host health

The human gut microbiota (GM) is a complex and dynamic ecosystem that plays a pivotal role in benefiting human health and overall well-being. It maintains host physiological homeostasis through diverse host-microbe interactions, including immune modulation, antagonistic inhibition of pathogen colonization, and facilitation of nutrient absorption. Factors such as diet, antibiotic use, age and host genomics all contribute to the initiation and maintenance of GM diversity, with diet recognized as the most influential determinant. Dietary interventions can modulate the abundance of various bacterial species in the gut, thereby shaping health outcomes, through the consumption of dietary fiber, probiotics, prebiotics, synbiotics, etc. The aim of this Research Topic is to explore the molecular mechanisms of host-microbe interactions and investigate how dietary interventions influence human health and disease through the modulation of the GM.

In this Research Topic, we begin by presenting research focused on diabetes. As is well known, diabetes is a chronic metabolic disorder marked by elevated blood glucose levels, intricately linked to metabolic dysregulation and systemic inflammation. Impaired glucose tolerance (IGT) is a precursor state of diabetes. To explore the complex interaction between GM, IGT, and diabetes, Zhen et al. first obtained 465 microbiome-related metagenomic samples from the NCBI (National Center for Biotechnology Information) database, including 225 samples from individuals with type 2 diabetes and 240 samples from healthy controls, to identify the key genera and associated functional pathways of diabetes. Furthermore, stool and blood samples were collected from 49 individuals with IGT and 27 healthy volunteers. Multi-omics approaches were then employed to analyze gene expression changes in the GM and its associated metabolites in these samples. The experimental results indicate that throughout the progression from IGT to diabetes, the GM undergoes dysbiosis, characterized by an expansion of *Blautia* and a reduction in *Faecalibacterium*, leading to decreased levels of tauroursodeoxycholic acid (TUDCA) and carnosine (CARN). Subsequent cell experiments confirmed that lower levels of TUDCA and CARN induced increased expression of insulin-like growth factor-binding protein 3 (IGFBP-3) and interleukin-6 (IL-6) in HepG2 cells and neutrophils, exacerbating inflammation and promoting hyperglycemia. These results suggest exogenous modulation or supplementation of GM components or their derived metabolites may represent a novel approach for the prevention and treatment of diabetes. In addition, gestational diabetes mellitus (GDM) is a type of diabetes that develops only during pregnancy. Latest studies have shown that the GM is closely associated with the GDM. To gain a deeper understanding of its role, Ma et al. reviewed the research on the interaction between GM and GDM, elucidating the molecular mechanisms by which the microbiota contributes to the onset and progression of GDM. They highlighted the significance of interventions such as fecal bacteria transplantation (FMT), probiotics, and prebiotics in the prevention and treatment of GDM, offering new avenues for its management.

Ulcerative colitis (UC) is a gastrointestinal disorder marked by chronic inflammation of the colonic mucosa. Conventional drug treatments for UC often lead to local or systemic side effects, significantly reducing patients’ quality of life. To address this challenge, exploring alternative therapies for UC is of paramount importance. Recent studies suggest that the microbiome may play a therapeutic role in UC management. In this context, Jadhav et al. provide a comprehensive review of the pathological impact of GM dysbiosis in UC. They also propose microbiome-based therapeutic strategies aimed at restoring microbial balance and improving clinical outcomes, with particular emphasis on the potential of probiotics, prebiotics, and synbiotics. These interventions offer innovative strategies for controlling the GM, reducing inflammation, and improving overall health, providing more effective and safer treatment options for UC patients.

Osteoarthritis (OA) is a degenerative joint disease characterized by the progressive breakdown of cartilage, impaired joint function, and joint inflammation. Emerging evidence suggests that dietary fiber can alleviate OA by modulating the GM. However, the underlying gut-bone connection mechanisms remain incompletely understood. Using the anterior cruciate ligament transection (ACLT) method to establish an OA model in Sprague-Dawley (SD) rats, Wu et al. conducted an 8-week dietary intervention with varying levels of dietary fiber. The results revealed that high dietary fiber (HDF) intake increased the abundance of *Bacillota* as the dominant microbiota, enhanced the expression of tight junction proteins to reduce intestinal permeability, and upregulated the expression of Sestrin2 (SESN2) in the knee joint. This maintained chondrocyte activity, reduced inflammation, and alleviated the severity of osteoarthritic changes, supporting dietary interventions that modulate the GM as a potential therapeutic approach for OA.

Childhood is a critical stage of growth and development, during which the GM plays a vital and irreplaceable role in maintaining health. However, the composition of the GM shows a strong geographical correlation, stressing the importance of addressing research gaps in different regions. Yang et al. collected fecal and blood samples from 100 healthy children aged 2 to 12 years from Northwest China and used shotgun metagenomic sequencing as well as untargeted metabolomics to analyze the composition of the gut microbiota and metabolites. The results revealed that factors such as age, body mass index (BMI), regular physical activity, and delivery mode significantly influenced the diversity of the intestinal microbiota and metabolite levels, highlighting the region-specific gut microbiome characteristics. The research provides important insights and a reference for healthcare in children from the northwest region of China, and contributes significantly to advancing research on Host-Microbe Interactions.

Overall, the five articles on this Research Topic reveal the important role of dietary interventions in regulating host-microbe interactions, offering us a novel perspective on maintaining health and treating diseases. However, the complex mechanisms underlying dietary interventions in regulating host-microbe interactions still require further exploration. In the future, integrating multi-omics data (such as genomics, transcriptomics, and metabolomics) will help comprehensively unveil the intricate relationship between diet and host-microbe interactions, advancing the application of dietary interventions in health management.

